# Surveillance of *Plasmodium vivax* transmission using serological models in the border areas of China–Myanmar

**DOI:** 10.1186/s12936-022-04096-8

**Published:** 2022-03-03

**Authors:** Meixue Yao, Lishun Xiao, Xiaodong Sun, Zurui Lin, Xiao Hao, Qiong-qiong Bai, De-Hui Yin

**Affiliations:** 1 Key Laboratory of Environment and Health, Department of Epidemiology and Health Statistics, School of Public Health, Xuzhou Medical University, No.209, Tongshan Road, Xuzhou, 221004 Jiangsu China; 2 Yunnan Institute of Parasitic Diseases, No.6 Xiyuan Road, Simao City, Puer, 665000 Yunnan China; 3 Jinan Blood Center, No.127 Jingliu Road, Jinan, 250001 Shandong China

**Keywords:** *Plasmodium vivax*, Surveillance, Serology, Seroconversion, Rate, Transmission intensity, China–Myanmar border

## Abstract

**Background:**

To understand the *Plasmodium vivax* malaria transmission intensity and to assess the effectiveness of prevention and control measures taken along the China–Myanmar border, a catalytic model was used to calculate the seroconversion rate, an important indicator of malaria transmission intensity with high sensitivity, which is particularly useful in areas of low transmission.

**Methods:**

Five counties in Yunnan Province bordering Myanmar were selected as survey sites, and subjects were obtained in each county by stratified random sampling in 2013–2014. Fingerstick blood was collected from each subject and tested for antibodies to *P. vivax* Merozoite Surface Protein 1-19 (PvMSP1-19) using indirect ELISA. A catalytic conversion model was used to assess the transmission intensity of *P. vivax* malaria based on the maximum likelihood of generating a community seroconversion rate.

**Results:**

A total of 3064 valid blood samples were collected. Antibody levels were positively correlated with age. The seroconversion rate (SCR) values for each village were Luoping (0.0054), Jingqiao (0.0061), Longpen (0.0087), Eluo (0.0079), Banwang (0.0042) and Banbie (0.0046), respectively.

**Conclusion:**

Overall, the intensity of *P. vivax* malaria transmission in the border areas of Yunnan Province is low and not entirely consistent across counties. Consecutive serological surveys are needed to provide a sensitive evaluation of transmission dynamics and can help to confirm areas where infection is no longer present.

## Background

Since the Chinese government launched the National Malaria Eradication Programme (NMEP) in 2010, China has made great progress in malaria control [1, 2], with a significant decrease in malaria incidence [3], and most regions have eliminated malaria. However, Yunnan Province is located in the Greater Mekong River Basin, where malaria is highly prevalent [4]; it has extensive borders with Myanmar, Laos, and Vietnam, and there are frequent population movements across the borders in these areas [5], which increases the difficulty of malaria elimination in the region [6]. In addition, the border area of Yunnan has a special geographical environment and complex climate, and there are many different of malaria vectors [7, 8]. The dominant species are *Anopheles sinensis* and *Anopheles liangshanensis*, followed by *Anopheles kunmingensis*, *Anopheles minimus* and others, of which *An. kunmingensis* is an efficient vector (its malaria transmission effect is 110 times that of *An. sinensis*) [9]. Therefore, there is a need to strengthen malaria surveillance in Yunnan Province, especially for *P. vivax* malaria, which is predominant in the region.

For areas with low intensity malaria transmission, traditional surveillance methods such as parasite prevalence and entomological inoculation rates (EIRs), of which the EIR is considered the gold standard for assessing malaria transmission intensity, are no longer applicable due to their low sensitivity [10, 11]. Unlike many other infectious diseases, malaria antibodies against parasite antigens are widely divergent and some may last for a longer time than others [12, 13]. Antibody status may not be suitable for diagnostic purposes [14], but serology has been proposed as a sensitive and reliable tool for evaluating the level of immunity and the intensity of malaria transmission in populations, and it is particularly suitable for areas with very low malaria transmission or areas in the early eradication phase due to its high sensitivity [10, 15–18].

Given that malaria antibodies exhibit complexity in nature, resulting from species-, stage- and strain-specific antigenic diversity [19–21], whether an appropriate serological marker can be selected is the critical core of this method. Various malaria antigens have been used as serological markers for *P. vivax* malaria [22], and seroepidemiology and serokinetics of PvMSP1-19, PvDBPII and PvAMA1 were assessed to evaluate their usefulness as serological markers for the local transmission of malaria [23]. The high polymorphism in the PvAMA1 gene affected the antigen-specific response, limiting the role of PvAMA1 as a serological marker [24]. PvDBPII is not suitable as a serological marker to assess local transmission of malaria due to its persistent antibody status and potential as a vaccine candidate. Antibodies against PvMSP1-19 were found to be stable, with antibodies against MSP1-19 observed no more than 9 months after infection, suggesting that it could be used as a serological marker to track local transmission of malaria in a low transmission setting. In addition, there was no cross-reactivity between all four common *Plasmodium* species for PvMSP1-19 antibodies [25].

Few data on the serological surveillance of *P. vivax* in the border areas of Yunnan are available; therefore, PvMSP1-19 was used as the serological marker in this study to evaluate the transmission intensity of *P. vivax* in Yunnan’s border areas, to understand the prevalence of *P. vivax* in Yunnan’s border areas, to provide basic information for malaria prevention and control measures in these areas, and to supplement data for the malaria serological surveillance database.

## Methods

### Study sites, subjects and sample collection

Yunnan Province is located in southwestern China; it shares a 4060-km-long border with its neighbors Myanmar, Laos, and Vietnam. The border between China and Myanmar is 1997 km. Based on the 2012 summary report of malaria prevention and control in Yunnan Province, five of the top ten counties in terms of malaria incidence were selected for inclusion in this study, namely, Tengchong, Yingjiang, Ruili, Gengma and Menglian, of which Tengchong, Yingjiang and Ruili were the areas with the highest incidence for three consecutive years, from 2011 to 2013 [26].

The study population was collected from the beginning of 2013 to the end of 2014 using stratified random sampling, with 1–2 villages selected in each county, and participants were required to be at least 2 years old and to have lived in the survey area for at least 3 months. Fingerstick blood samples were obtained using uniform Whatman 903 filter paper, requiring a volume of no less than 100 µl of blood per drop and a blood spot diameter greater than 8 mm. Filter paper blood slices were dried naturally and clearly marked with a number, placed in self-sealing bags, one per person, and then adequate desiccant with color indication was added. Filter paper blood slices were stored at 20 °C for short-term storage and at − 80 °C for long-term storage. It was necessary to check the colour change of the desiccant periodically. If the desiccant changed from blue to pink, the seal of the bag had to be checked, and new desiccant had to be added in a timely fashion. One hundred healthy volunteer donors from Jinan, China, who had never been exposed to malaria were chosen as non-epidemic controls. All blood samples were diagnosed by RNA hybridization assays as described previously [27].

The sample size was calculated using a standard formula for prevalence studies as follows:

$$\mathbf{n}=\frac{{Z}^{2}P(1-P)}{{d}^{2}}$$

where n is the sample size and Z is a Z statistic value of 1.96 at a confidence level of 95%. P is considered prevalence at 12% [28], and d is a 3% relative precision. Ten percent of the calculated sample size was added to account for missing samples. The sample size was 495 for each district.

### Antibody detection

Anti-PvMSP1-19 antibodies were detected by indirect ELISA methods using the *P. vivax* antibody kit produced by Yisimeiquan Biotechnology (Shanghai) Co., Ltd., batch number: 20140701, which was supervised by the Institute of Parasite Prevention and Control of the Chinese Center for Disease Control and Prevention.

All filter paper blood samples were tested for antibody levels according to the instructions of the kit. Briefly, paper blood samples were taken 4 times with a 3 mm punch (once with 903 filter paper), dissolved in 300 µl of sample diluent and mixed uniformly at 100 rpm with a stirrer at room temperature. Positive controls were diluted into standard curves according to gradients of 1:25, 1:50, 1:100, 1:200, 1:400 and 1:800. The negative control was diluted at 1:20. Control and blood spot eluates were added in duplicate (100 µl) and incubated at 37 °C for 1 h. Next, the plates were washed and incubated with the enzyme conjugate at 37 °C for 1 h. After washing, a developing solution was added and the reaction was terminated after 10 min. The optical density (OD) was measured at 450 nm. Each plate contained a negative control, a blank control (sample dilution) and a standard curve control at a 1:25–1:800 dilution. A standard curve was established by fitting a logarithmic curve with the concentration (x) and OD (y) of the reference substance to obtain the parameters a and b of the mathematical formula. The antibody concentration of each sample was calculated according to the given formula.

### Data processing and statistical analysis

The mean titer of the non-epidemic negative control group plus 3 times the standard deviation was used as the cutoff value to classify the anti-malarial antibody results in the sera of residents in the Yunnan border counties as either negative or positive. The ratio of antibody concentration to cutoff value for residents of the Yunnan border areas is recorded as OD%. Descriptive statistics were used to show basic information of the participants. The chi-square test was used to compare the differences between qualitative data. Differences between the antibody concentrations of the two groups were analyzed using the Mann–Whitney test. Spearman’s rank correlation test was used to test the relationship between antibody levels and age. In epidemic areas, the level of anti-malarial antibodies in serum is dynamically changing, where the probability of positive to negative change is called the seroreversion rate, while the probability of negative to positive change is called the seroconversion rate. A reversible catalytic model was fitted to the dichotomized data using the maximum likelihood method [29]. The model generated a seroconversion rate (SCR or $\uplambda$) and a seroreversion rate ($\uprho$). The equation fitted was as follows [11]:

$${\mathrm{P}}_{\mathrm{t}}=\frac{\uplambda }{\uplambda +\uprho }(\left(1-\mathrm{exp}(-(\uplambda +\uprho )\mathrm{t}\right))$$

Statistical analyses were performed using IMB SPSS statistics 21 for Windows, and the above models were fitted using R software. P values less than 0.05 were considered significant.

### Ethical approval

Ethical approval was received from the Ethics Committee of Xuzhou Medical University. Informed consent was obtained from all participants, and from guardians for children < 18 years of age.

## Results

### Study participants

**Fig. 1:**
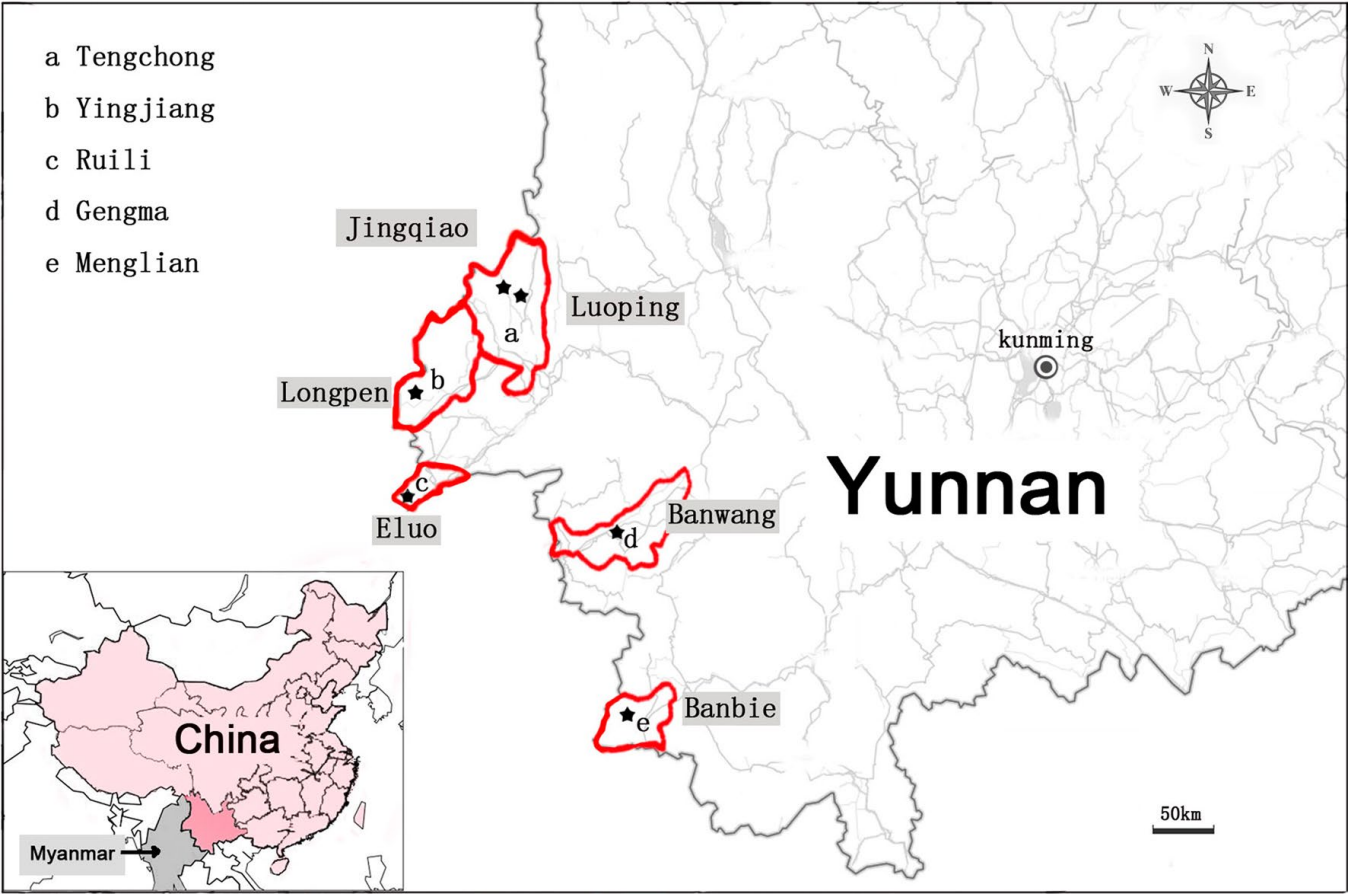
The specific locations of the villages selected in this study

Six villages in five counties in Yunnan Province bordering Myanmar were selected as the survey sites: Luoping and Jingqiao in Tengchong County, Longpen in Yingjiang County, Eluo in Ruili County, Banwang in Gengma County and Banbie in Menglian County, the specific locations of which are shown in Fig. 1. A total of 3064 valid blood samples were collected, including 504 (16.4%) from individuals in Luoping village, 529 (17.3%) from individuals in Jingqiao village, 546 (17.8%) from individuals in Longpen village, 553 (18.0%) from individuals in Eluo village, 620 (20.2%) from individuals in Banwang village and 312 (10.3%) from individuals in Banbie village. There was no significant difference in the sex ratio among individuals from the villages (*P* > 0.05). A total of 4 cases of *P. vivax* malaria infection were found, including 3 cases in Eluo and 1 case in Longpen, and no *P. falciparum* malaria infection was found in any of the samples. The samples from each village were obtained from subjects in different age groups, as detailed in Table 1.

**Table 1:** The basic information of survey participants

	Tengchong		Yingjiang	Ruili	Gengma	Menglian
	Luoping	Jingqiao	Longpen	Eluo	Banwang	Banbie
**Gender n (%)**						
Male	220 (43.7)	220 (41.6)	258 (47.3)	201 (36.3)	301 (48.5)	122 (39.1)
Female	284 (56.3)	309 (58.4)	288 (52.7)	352 (63.7)	319 (51.5)	190 (60.9)
**Age n (%)**						
< 15	206 (40.9)	234 (44.2)	260 (47.6)	230 (41.6)	208 (33.5)	32 (10.3)
15–25	31 (6.2)	17 (3.2)	39 (7.1)	49 (8.9)	205 (33.1)	60 (19.2)
26–35	34 (6.7)	43 (8.1)	62 (11.4)	56 (10.1)	42 (0.068)	37 (11.9)
36–45	54 (10.7)	73 (13.8)	72 (13.2)	69 (12.5)	73 (11.8)	72 (23.1)
46–55	64 (12.7)	58 (11.0)	49 (9.0)	75 (13.6)	38 (6.1)	47 (15.1)
> 55	115 (22.8)	104 (19.7)	64 (11.7)	74 (13.4)	54 (8.7)	64 (20.5)
**Infection**						
pf	0	0	0	0	0	0
pv	0	0	1	3	0	0

### Anti-malarial antibody concentrations in different areas and at different ages

**Fig. 2:**
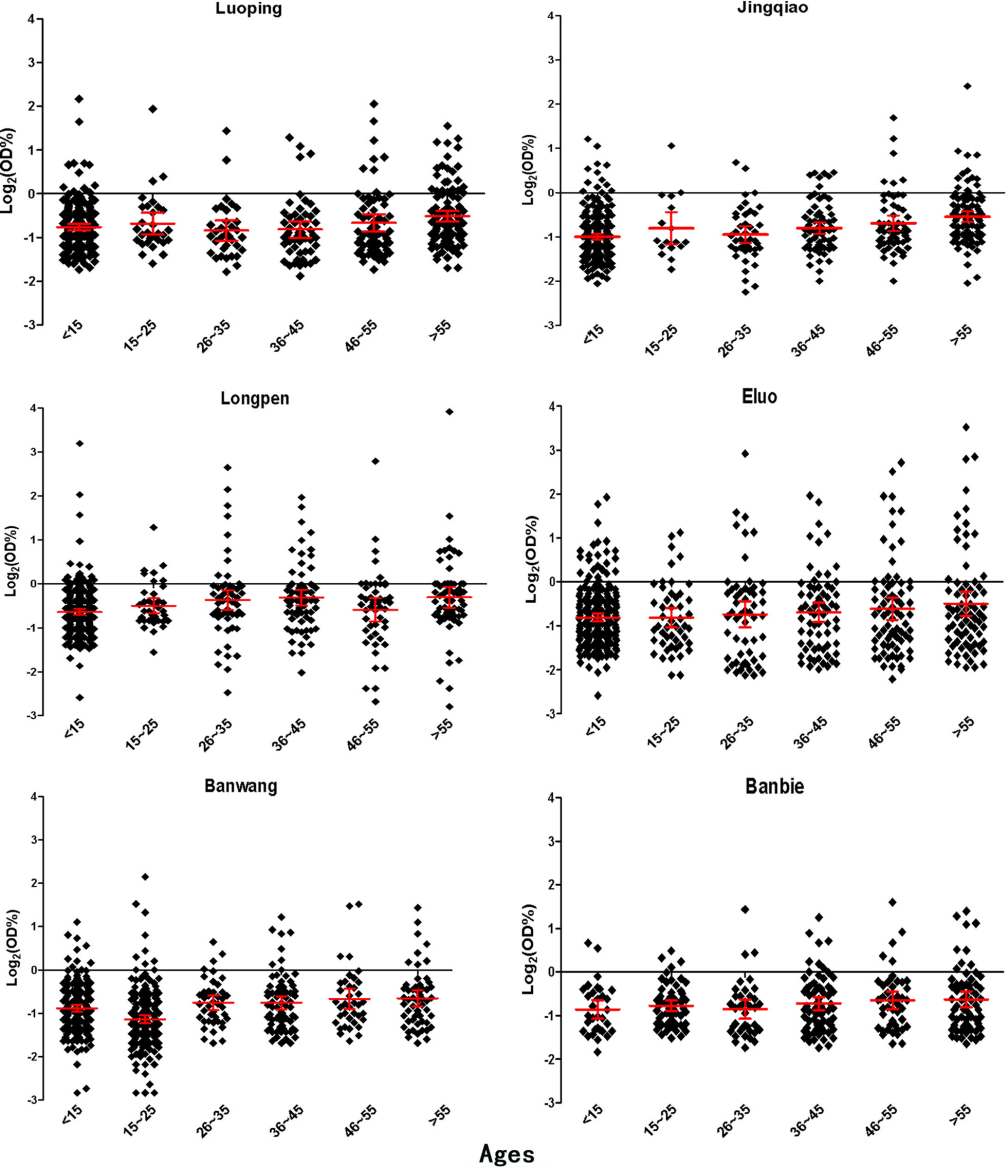
Levels of anti-malarial antibodies in different age groups at different study sites

The concentrations of antibodies to PvMSP1-19 were detected from the eluates of the blood samples collected from individuals in all the villages. Except for Longpen (P > 0.05), the results from the other five villages showed a positive correlation between antibody concentration and age with the following correlation coefficients: Luoping (r = 0.148, P = 0.001), Jingqiao (r = 0.287, P < 0.001), Eluo (r = 0.121, P = 0.004), Banwang (r = 0.194, P < 0.001) and Banbie (r = 0.143, P = 0.011); the detailed results were shown in Fig. 2. No significant difference was found in antibody concentrations between males and females in any of the villages tested (*P* > 0.05).

### Seropositive rate in each village

**Fig. 3:**
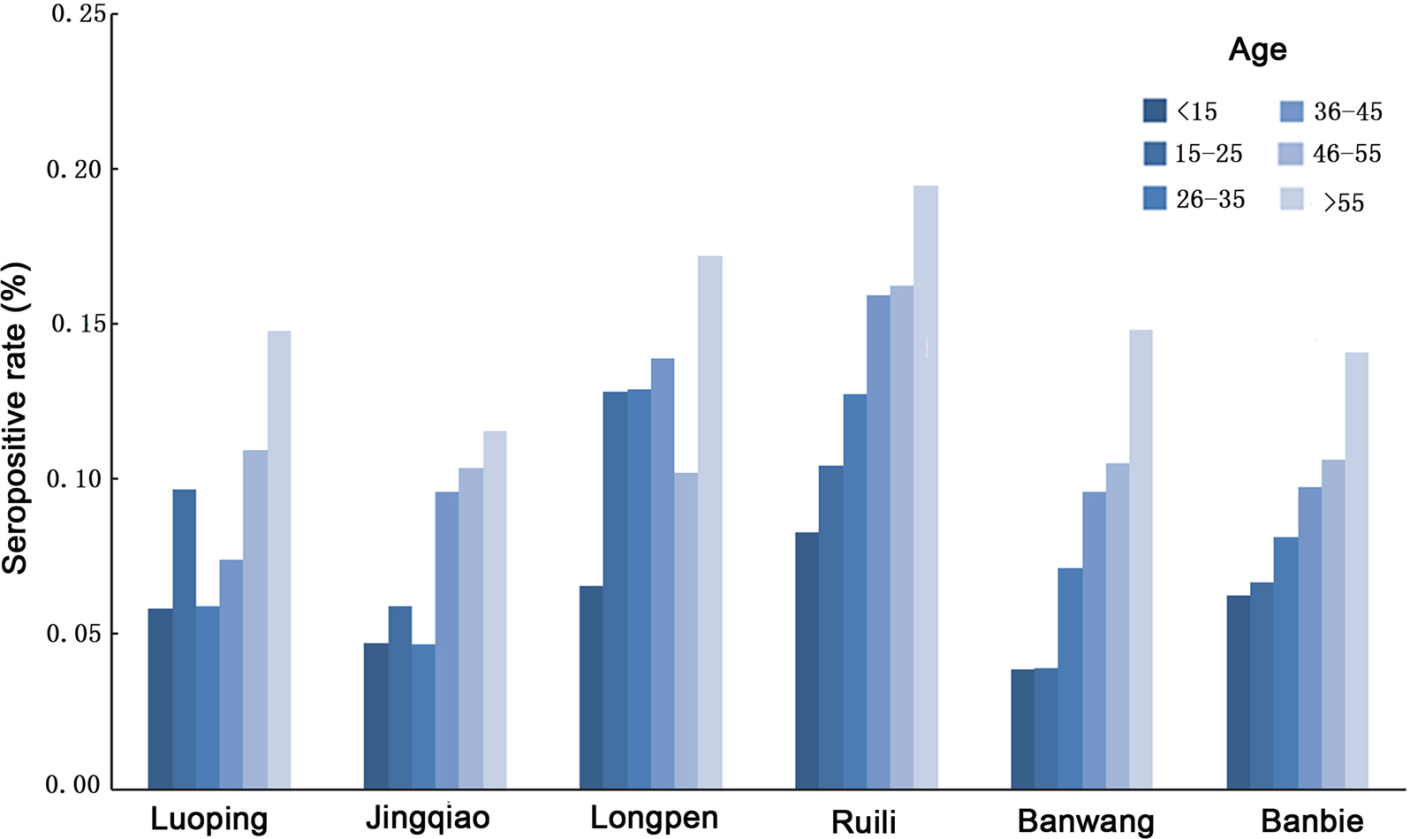
Seroprevalence rate in different age groups at different study sites

The highest total seropositivity rate was found in Eluo, with 12.41% (9.66–15.16%), followed by Longpen, with 10.26% (7.71–12.81%), and then in Banbie, Luoping, Jingqiao and Banwang, with seropositivity rates of 9.62% (6.35–12.89%), 8.92% (6.43–11.41%), 7.37% (5.14–9.60%), and 6.13% (4.24–8.02%), respectively. After stratification by age, the seropositivity rates obviously increased with age, as shown in Fig. 3.

### Seroconversion rate in each village

**Fig. 4:**
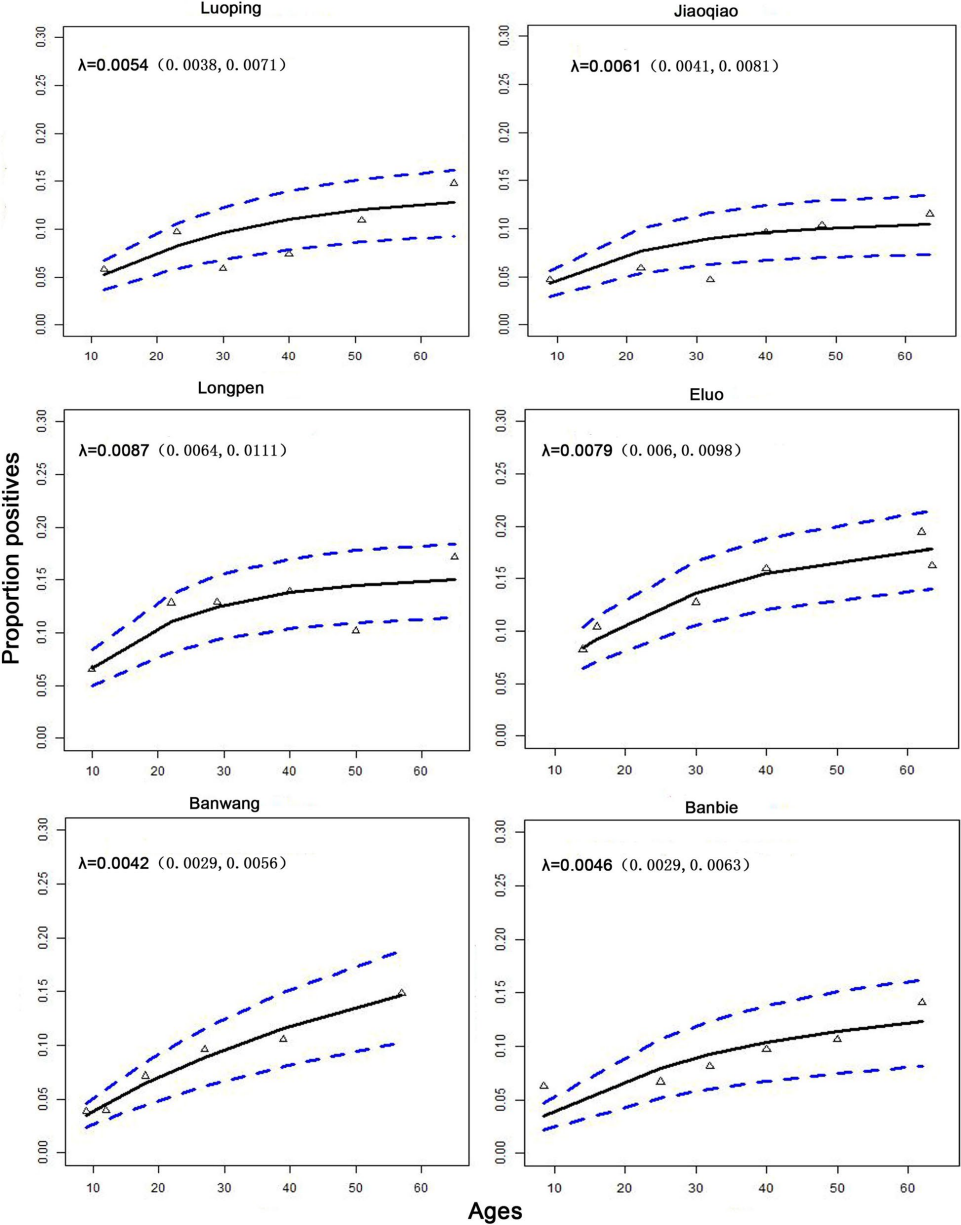
Seroprevalence curves for *P. vivax* antigens for each study sites. Hollow triangles in each graph represent age seroprevalence by decile. Solid lines represent maximum likelihood curves and broken lines 95% confidence intervals

SCR values and their 95% confidence intervals were calculated for individuals in each village using the reversible catalytic model with the following results: Luoping (0.0054, 95% CI 0.0038–0.0071), Jingqiao (0.0061, 95% CI 0.0041–0.0081), Longpen (0.0087, 95% CI 0.0064–0.0111), Eluo (0.0079, 95% CI 0.006–0.0098), Banwang (0.0042, 95% CI 0.0029–0.0056) and Banbie (0.0046, 95% CI: 0.0029–0.0063), as detailed in Fig. 4 and Table 2.

**Table 2:** The values of seroconversion and seroreversion rates in survey sites

Sites	Λ (95%CI)	Ρ (95%CI)
Luoping	0.0054 (0.0038–0.0071)	0.034 (0.0157–0.0517)
Jingqiao	0.0061 (0.0041–0.0081)	0.051 (0.0263–0.0755)
Longpen	0.0087 (0.0064–0.0111)	0.048 (0.0265–0.0694)
Eluo	0.0079 (0.0060–0.0098)	0.033 (0.0180–0.0482)
Banwang	0.0042 (0.0029–0.0057)	0.015 (− 0.0067 to 0.0362)
Banbie	0.0046 (0.0029–0.0063)	0.028 (0.0077–0.0483)

## Discussion

Due to the implementation of a robust surveillance and response system, of which the “1-3-7” surveillance approach is representative (cases are reported within 1 day, investigated within 3 days, and focused investigation and action is taken within 7 days [30, 31]), the number of malaria cases in China has declined dramatically. The number of reported malaria cases in Yunnan Province in 2014 was 533, a decrease of 65.0% compared to the 1522 cases reported in 2011. Among them, the number of reported cases of local infection was 51, a decrease of 86.0% compared with the 429 cases reported in 2011 [26, 32, 33]. Only four *P. vivax* cases were detected in this study, indicating that the malaria epidemic situation in border areas is well controlled. From 2012 to 2014, a total of 1 558 cases of malaria were reported in the 20 border counties of Yunnan Province, with a 41.03% decrease in incidence from the 680 cases reported in 2012 to 401 cases reported in 2014 [34].

In this study, both antibody concentrations and seroprevalence rates were found to increase with age. Residents living in epidemic areas are stimulated to produce a certain amount of protective antibodies due to their long-term exposure to malaria environments, and the level of anti-malarial antibodies gradually accumulates over time [11]. Malaria antibody levels are higher in the older age group, and this age-specific antibody response reflects cumulative exposure or differences in exposure related to behaviour [35].

Among these villages, the SCR values obtained using age-stratified seroprevalence ranged from 0.0042 to 0.0087, with relatively high values for Longpen in Yingjiang and Eluo in Ruili, indicating that these two border counties are at relatively high risk of malaria transmission and should strengthen malaria control measures. Both Ruili and Yingjiang share a long border with Myanmar, with no natural barriers and dozens of natural points of crossing. Frequent changes in the flow of people across the border make the exchange of infectious sources and mutual import and export prolific, which intensifies the spread and prevalence of malaria on both sides of the border and increases the difficulty of malaria prevention and control work. However, the SCR values obtained in this study are much lower than the SCR values for *P. vivax* reported in previous studies in other countries, where the serum markers used were also PvMSP-1-19. In two studies conducted in Cambodia, one obtained SCR values of 0.004–0.184 in 2005 [28], and the other obtained SCR values of 0.011 in 2012 [36] (over a similar timeframe as the present study). The SCR values of 0.005 to 0.041 obtained from serological monitoring in Vanuatu in 2009 [37] were also higher than those obtained from this study. This result suggests that there are significant differences in the intensity of malaria transmission between the areas in this study and other areas previously reported, and these differences reflect the effectiveness of malaria control measures in the region.

However, Yunnan Province still faces many challenges in eliminating malaria. First, although the number of local infections has decreased significantly, the local natural environment and transmission vectors have not changed fundamentally, and *An. sinensis* and *Anopheles microcephalus* are still important local transmission vectors and may cause secondary transmission during the transmission season once the source of infection is imported. In addition, the frequent cross-border movement of populations in the border areas of Yunnan Province, which are adjacent to countries with severe malaria endemicity, such as Myanmar has become one of the main factors affecting the achievement of China’s malaria elimination goals in Yunnan Province. Therefore, in the era of malaria (pre-)elimination, surveillance is critical to estimate local transmission, and highly sensitive serological surveillance methods can undoubtedly play an important role. Surveillance is not a singular event; rather, it is a long-term, continuous, systematic process. Regular surveillance helps to track malaria transmission dynamics over time and can help to confirm areas where infection is no longer present. This study provides serological information on the intensity of malaria transmission in the border areas of Yunnan, filling a lack in serosurveillance information in the region. It also provides data to support the evaluation and implementation of local malaria prevention and control measures, and it provides a comparative reference for malaria serological surveillance studies that have been or will be conducted in other regions.

## Limitation

An implicit assumption of a reversible serocatalytic model is that there is constant transmission over time and across all age groups, which is difficult to meet in an area pursuing malaria elimination; thus, choosing younger age groups as samples would be helpful. A drawback of this study is the large age span represented in the collected samples, which was due to the limitations of the demographic composition of the collection sites. In addition, the catalytic model is not infallible; one limitation is that it does not allow people to increase their seropositivity due to exposure when they are already seropositive [38], and another limitation is that a fixed cutoff needs to be imposed to distinguish between seropositive and negative individuals, so that continuous changes in antibody levels are ignored, thus potentially reducing the precision of the estimates [39].

## Conclusion

The low transmission intensity of *P. vivax* at the Yunnan border suggests that control measures have been successful over the years; however, monitoring is a long-term, continuous, systematic process in areas of low transmission intensity. Consecutive serological surveys help investigators gain insight into spatiotemporal patterns of malaria transmission, and the analysis of longitudinal serological data enables a sensitive evaluation of transmission dynamics. Considering that catalytic models may underestimate the realistic transmission intensity, it is still important to strengthen malaria surveillance in the region and to aim for more accurate models to assess transmission intensity in the future.

## Data Availability

Data sharing is not applicable as no datasets were generated or analyzed in this paper.
